# Perspectives for microbeam irradiation at the SYRMEP beamline

**DOI:** 10.1107/S1600577521000400

**Published:** 2021-02-15

**Authors:** Elisabeth Schültke, Stefan Fiedler, Ralf Hendrik Menk, Felix Jaekel, Diego Dreossi, Katia Casarin, Giuliana Tromba, Stefan Bartzsch, Stephan Kriesen, Guido Hildebrandt, Fulvia Arfelli

**Affiliations:** a Department of Radiooncology, Rostock University Medical Center, Südring 75, 18059 Rostock, Germany; b European Molecular Biology Laboratory, Notkestrasse 85, 22607 Hamburg, Germany; c Elettra-Sincrotrone Trieste, Strada Statale 14, Trieste 34149, Italy; d University of Saskatchewan, Saskatoon, Saskatchewan, Canada; e Trieste Section, Istituto Nazionale Fisica Nucleare (INFN), Trieste, Italy; f Department of Radiooncology, Technical University Munich, Munich, Germany; g Institute for Innovative Radiotherapy, Helmholtz-Zentrum Munich (HMGU), Munich, Germany; h Department of Physics, University of Trieste, Trieste, Italy

**Keywords:** microbeam irradiation, murine melanoma cells, microbeam, radiotherapy

## Abstract

It has been shown previously both *in vitro* and *in vivo* that microbeam irradiation (MBI) can control malignant tumour cells more effectively than the clinically established concepts of broad beam irradiation. With the aim to extend the international capacity for microbeam research, the first MBI experiment at the biomedical beamline SYRMEP of the Italian synchrotron facility ELETTRA has been conducted. Using a multislit collimator produced by the company TECOMET, arrays of quasi-parallel microbeams were successfully generated with a beam width of 50 µm and a centre-to-centre distance of 400 µm. Murine melanoma cell cultures were irradiated with a target dose of approximately 65 Gy at a mean photon energy of ∼30 keV with a dose rate of 70 Gy s^−1^ and a peak-to-valley dose of ∼123. This work demonstrated a melanoma cell reduction of approximately 80% after MBI. It is suggested that, while a high energy is essential to achieve high dose rates in order to deposit high treatment doses in a short time in a deep-seated target, for *in vitro* studies and for the treatment of superficial tumours a spectrum in the lower energy range might be equally suitable or even advantageous.

## Abstract 2

The first microbeam irradiation study at the biomedical beamline SYRMEP of the Elettra-Sincrotrone Trieste, the technical setup of the experiment and the results of the associated cell culture study in murine melanoma cells are reported.

## 1. Introduction   

Microbeam irradiation (MBI) with therapeutic intent has become known as microbeam radiotherapy (MRT). During the last three decades, more than 100 publications have been registered in the Pubmed database, reporting on the development of specialized equipment for microbeam irradiation as well as on *in vitro* and *in vivo* experiments assessing the potential of MRT for the treatment of malignant and non-malignant diseases (Schültke *et al.*, 2017 ▸). MBI is an experimental technique using spatial dose fractionation at the micrometre range. The X-ray beam generated by a synchrotron storage ring is split by a collimator into an array of quasi-parallel microbeams ≤100 µm full width at half-maximum (FWHM). Thus, an irradiation field with a repetitive peak and valley dose pattern is created. A high peak-to-valley dose ratio (PVDR), created by the prominent peak dose deposited in the paths of the microbeams and the valley dose zones between the paths of the microbeams, is essential for the preservation of normal tissue function (Siegbahn *et al.*, 2006 ▸; Serduc *et al.*, 2009 ▸). The high photon flux of a synchrotron provides the required high peak dose rate to preserve a steep dose gradient at the microbeam edges. The steep dose gradient is important because it guarantees relatively low valley zone doses, corresponding to high normal tissue tolerance. Fast administration of the target dose is especially important when irradiating a living and thus potentially pulsating or moving tissue in order to prevent smearing of the microbeam edges.

MBI experiments have been conducted at the NSLS at Brookhaven National Laboratories (Slatkin *et al.*, 1995 ▸; Laissue *et al.*, 1998 ▸) at SPring-8 (Crosbie *et al.*, 2010 ▸) and at the Australian Synchrotron (Livingstone *et al.*, 2017 ▸). In Europe, until recently MBI studies were conducted exclusively at the biomedical beamline ID17 of the European Synchrotron Radiation Facility (ESRF) in Grenoble, France. Over the last decade, to support a timely transition into clinical microbeam radiotherapy trials, there has been a steadily increasing demand for beam time to conduct microbeam experiments.

Therapeutic efficacy has been demonstrated in several small-animal models of malignant brain tumours (Laissue *et al.*, 1998 ▸; Miura *et al.*, 2006 ▸; Bouchet *et al.*, 2016 ▸; Schültke *et al.*, 2018 ▸; Engels *et al.*, 2020 ▸). The results of other studies have shown evidence of a remarkably high normal-tissue tolerance (Laissue *et al.*, 2007 ▸; Schültke *et al.*, 2008 ▸; Bouchet *et al.*, 2010 ▸).

With an increasing spectrum of malignant tumours, which could potentially benefit from MBI, it seems reasonable to explore the opportunities to conduct microbeam experiments also at other synchrotron beamlines. Especially for the treatment of superficial tumours like melanoma and osteosarcoma, treatment at beamlines with lower critical energies might even be favourable because one could take advantage of the build-up effect described for orthovolt radiotherapy (Hill *et al.*, 1998 ▸).

We have designed a pilot experiment to test whether microbeam arrays suitable for biomedical research can be conducted at the biomedical beamline SYRMEP of the Elettra-Sincrotrone Trieste, Italy, with a view on providing instrumental and methodological development for future clinical MRT trials at the ESRF and the Australian Synchrotron.

## 2. Material and methods   

### 2.1. Technical setup of the experiment   

The MBI experiment described here utilizes polychromatic synchrotron radiation generated by the bending magnet and has been carried out in the white/pink X-ray beam station for planar and computed micro-tomography of the SYRMEP beamline at Elettra-Sincrotrone Trieste (Tromba *et al.*, 2010 ▸). During the experiment the electron storage ring was operated at 2.4 GeV with a beam current of 160 mA. The magnetic field of the bending magnet was 1.45 T.

MBI relies on a custom-made multi-slit collimator (MSC) (provider Tecomet Subsidiary, Viasys Healthcare Inc., 170 New Boston Street, Woburn, MA 0180, USA), which was inserted into the hardware and software environment of the SYRMEP beamline. This collimator, containing two separately moveable 8 mm-deep tungsten lamellae to produce microbeam arrays with different beam widths and different centre-to-centre-distances, has been extensively characterized (Bräuer-Krisch *et al.*, 2005 ▸). The necessary control unit, which allows the user to shift and rotate the two collimator stacks against each other for internal alignment, was designed and produced at the workshop of the European Molecular Biology Laboratory (EMBL) in Hamburg, Germany.

**Figure 1:**
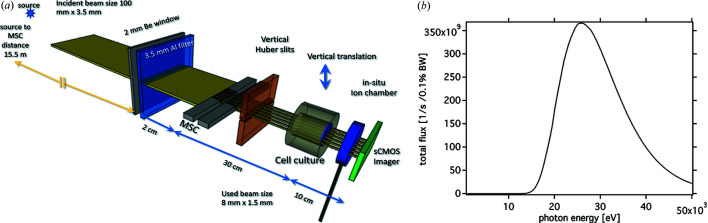
(*a*) Sketch (not to scale) of the MRT setup in the pink/white beam station. (*b*) Calculated filtered pink/white beam spectrum from the SYRMEP bending magnet operating at a magnetic field of 1.45 T, taking into account an electron beam energy of 2.4 GeV, a beam current of 160 mA and the two filters (2.0 mm beryllium and 3.5 mm aluminium).

The MSC was positioned in the polychromatic synchrotron beam on a motorized rotation stage downstream of a 2.0 mm-thick beryllium window, approximately 15 m downstream of the radiation source in an enclosure within the optics hutch of the SYRMEP beamline [Fig. 1 ▸(*a*)].

The MSC accepts 30 mm horizontally and 5 mm vertically of the incident polychromatic beam, generating a microbeam array of quasi-parallel microbeams with 50 µm beam width and a centre-to-centre distance of 400 µm.

Due to the incident horizontal beam divergence of 7 mrad, the microbeams obtained at the edges of the horizontal microbeam array became increasingly narrower. For this reason, we only used the 20 central microbeams for our experiment, corresponding to a horizontal width of 8 mm. In order to adjust the dose rate, an aluminium filter (3.5 mm Al) was inserted into the beam. The resulting energy spectrum of the polychromatic bending magnet radiation calculated utilizing the *XOP* toolkit (version 2.4; Sanchez & Dejus 2011 ▸) is shown in Fig. 1 ▸(*b*).

The samples were placed on a motorized stage that allows translations perpendicular to the beam direction (vertically and horizontally) of several centimetres with variable velocities. The distance between the collimator and the sample stage was 30 cm.

Furthermore, we used an sCMOS (scientific complementary metal-oxide semiconductor) imager (Orca Flash, Hamamatsu), which was permanently installed downstream of the sample stage area to record static images of the microbeam array. The detector is optically coupled to a 45 µm-thick GGG:Eu (Gd_3_Ga_5_O_12_:Eu) scintillator utilizing a set of optical lenses with different magnifications. The sCMOS sensor comprises 2048 × 2048 pixels and possesses a dynamic range of 37000:1. The highest magnification was used, which translates to a pixel size of 0.5 µm × 0.5 µm. The entire space along the beam path available within the white/pink beam station for the setup was approximately 1 m.

### 2.2. Simulation and dosimetry   

#### 2.2.1. Dose simulation   

Monte Carlo dose calculations were carried out in *Geant4* (version 10.3.3) using the low-energy physics Penelope libraries (Allison *et al.*, 2016 ▸) and applying the geometry as depicted in Fig. 1 ▸(*a*). *Geant4* is a well benchmarked Monte Carlo toolkit and has frequently been employed for dose calculations in MRT (Spiga *et al.*, 2007 ▸; Bartzsch *et al.*, 2014 ▸; Cornelius *et al.*, 2014 ▸). The MSC, horizontal and vertical slit apertures and cell culture vessel were included in the Monte Carlo model.

Photons were assumed to originate 15.5 m upstream of the MSC from a source with Gaussian position and angle profile. The width of these profiles was chosen such that the observed beam divergence of 0.175 mrad (vertical) and 7 mrad (horizontal) was met. The photon spectrum of the incident bending magnet radiation at the SYRMEP beamline was simulated using *XOP* (version 2.4; Sanchez & Dejus, 2011 ▸) for a bending magnet with a magnetic field of 1.45 T, an electron energy of 2.4 GeV and beam filtering of 2.0 mm beryllium and 3.5 mm aluminium [Fig. 1 ▸(*b*)]. The mean energy of the spectrum was approximately 30 keV.

Energy was scored in 5 µm × 100 µm-sized voxels in a 1 mm-thick water layer at the position of plated cells in the experiment. In total, the simulation tracked 3 × 10^10^ photon histories resulting in relative dose uncertainty in the peak of <1% and the valley of <5%. Scanning of the sample through the beam was simulated by virtually moving the source in 100 µm steps across the sample plane. Similar simulations have been performed for previous experiments (Serduc *et al.*, 2014 ▸; Fardone *et al.*, 2018 ▸; Bartzsch *et al.*, 2015 ▸).

#### 2.2.2. Absolute dosimetry   

Prior to the cell irradiation we conducted quantitative dosimetry on the incident beam with and without insertion of the MSC, utilizing two calibrated and commercially available dosimeters mounted at the sample position. One dosimeter was a microDiamond crystal (60019, PTW, Freiburg, Germany) and the other dosimeter was a partially depleted passivated implanted planar silicon detector (PIPS PD50-11-500AM, Canberra Industries Inc., Belgium). The latter has an active area of 50 mm^2^ and a thickness of 500 µm. The microDiamond is a synthetic single crystal with outer measurements of 7 mm × 45.5 mm, a circular sensitive area defined by a 1.1 mm radius and a nominal sensitive volume of 0.004 mm^3^. It is designed for operation with high voltage and the measurements are temperature-independent. For the readout, a Unidos electrometer (PTW, Freiburg, Germany) calibrated for use with the microDiamond was used. Since the absorption of both devices is not neglectable they had to be removed for the irradiation of the cell cultures. For *in situ* beam monitoring during cell irradiation, a custom-made, air-filled semitransparent ionization chamber similar to that described by Menk *et al.* (2007 ▸) was installed downstream of the cell cultures.

It is noteworthy that all three aforementioned devices are not suitable to provide any information about the horizontal dose distribution of the microbeams. During cell irradiation, the microbeams were also monitored in real time utilizing the sCMOS camera, which was placed downstream of the *in situ* ionization chamber (Fig. 1 ▸).

#### 2.2.3. Relative dosimetry   

For relative dosimetry, two types of X-ray sensitive Gafchromic film were used: EBT3 and HD-V2 Gafchromic films (Ashland, USA). According to the product specification sheets, the dynamic dose range of EBT3 film is 0.1–20 Gy and the spatial resolution is approximately 25 µm. The dynamic dose range of HD-V2 film is 10–1000 Gy and the spatial resolution is approximately 5 µm. Thus, the spatial resolution is sufficient for dosimetry of 50 µm-wide microbeams. The energy dependence of the film is considered minimal. Due to the different dynamic dose ranges and spatial resolution parameters of the films, one would expect that intensity variations across individual microbeams are most likely to be better detected with HD-V2 Gafchromic film while intensity variations across the valley region are better shown on EBT3 Gafchromic film.

Film dosimetry for microbeam irradiation has been described extensively elsewhere (Ocadiz *et al.*, 2019 ▸; Pellicioli *et al.*, 2019 ▸). In brief, the microbeam array with planar view was recorded as the intensity pattern generated by the collimator by moving the films with constant velocity vertically through the stationary beam utilizing the vertical movement stage. The exposed EBT3 and HD-V2 Gafchromic films were then photographed using a microscope equipped with a light-emitting diode source.

### 2.3. Sample irradiation   

#### 2.3.1. Melanoma cell cultures   

For a first assessment of the therapeutic efficacy achievable with MBI at SYRMEP, we irradiated plated adherent cultures of a commercially available rodent melanoma cell line (F10B16). The characteristics of this cell line include fast and aggressive growth and a high degree of radioresistance, which is also typical for the natural course of the disease in human patients. Non-irradiated cell cultures were used as controls.

Melanoma cells were seeded in 24 well plates, 2.000 cells per well. Two days after seeding, at approximately 70% confluency, they were submitted to irradiation at the SYRMEP beamline.

#### 2.3.2. Microbeam irradiation   

For irradiation, most of the growth medium was aspirated because the multi-well plates were mounted upright in the path of the beam. Only a thin film of fluid was present during irradiation. The cell cultures were irradiated with an array of quasi-parallel microbeams of 50 µm beam width and a centre-to-centre distance of 400 µm for the unidirectional monoplanar MBI technique (originating from one single port) in one single irradiation fraction. The peak dose was approximately 65 Gy, resulting in valley doses <1 Gy. Cell cultures were terminated and cells were counted at 24 h and at 72 h after MBI.

There were triplicates for all cell counts, including the non-irradiated controls. The samples to be irradiated were translated vertically through the beam using a pre-calculated speed, leading to an adaptable dose according to dose (on sample) = beam height/speed × dose rate. At the irradiation position, the beam height was 1.5 mm (FWHM), the vertical speed of the sample stage was set to 1.638 mm s^−1^ and the dose rate was approximately 70 Gy s^−1^.

## 3. Results   

### 3.1. Simulation and dosimetry   

As mentioned above, during cell irradiation the microbeams were also monitored in real time utilizing the sCMOS camera, which was placed downstream of the *in situ* ionization chamber (Fig. 1 ▸).

**Figure 2:**
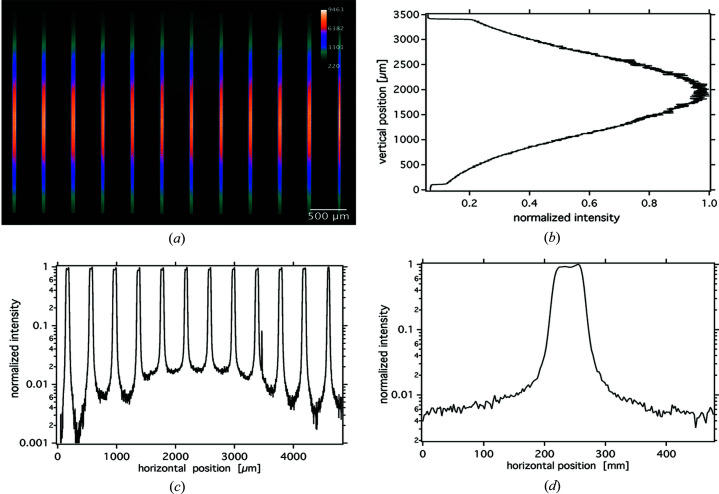
(*a*) Microbeam array recorded with sCMOS camera and (*b*)–(*d*) associated vertical and horizontal profiles.

A representative image of microbeam array, which has been acquired during MBI with the sCMOS camera, is depicted in Fig. 2 ▸(*a*). Since the field of view (FoV) of the sCMOS screen is limited to 6 mm and thus substantially smaller than the microbeam array, only the twelve central microbeams were recorded.

Shown in the upper right panel [Fig. 2 ▸(*b*)] is the vertical Gaussian intensity profile of the pink beam at SYRMEP featuring an FWHM of approximately 1.5 mm. The opening of the vertical slits as projected on the screen of the sCMOS imager was 3.265 mm. This vertical profile has been obtained by a horizontal averaging over 30 µm using one of the single microbeams in the centre of the image of the microbeam array [Fig. 2 ▸(*a*)]. Utilizing a vertical averaging over 500 µm yielded the horizontal intensity as presented in Fig. 2 ▸(*c*), showing the regular horizontal distribution across the individual microbeams in the array. Moreover, a 500 µm vertically averaged horizontal beam profile of a single microbeam is presented in Fig. 2 ▸(*d*) using the full spatial resolution of the sCMOS imager. The increased intensity at the right-hand side of the microbeam is probably the result of edge scattering or a parallax effect due to a slightly misaligned yaw axis of the MSC.

As mentioned before, a simplified system to represent the experimental setup was generated as the basis for the Monte Carlo calculations [Fig. 1 ▸(*a*)].

We assumed the height of the cell culture in one well of the 24-well plate to be equal to 1 mm and a vertical movement of the target through a beam with a vertical beam size of 1.5 mm (FWHM) possessing a Gaussian beam profile as depicted in Fig. 2 ▸(*b*). The energy spectrum [Fig. 1 ▸(*b*)] has been used as input for the simulations. The calculations were based on a total of 2.8 × 10^10^ photons and a beam divergence of 7 mrad × 0.175 mrad (horizontal × vertical).

**Figure 3:**
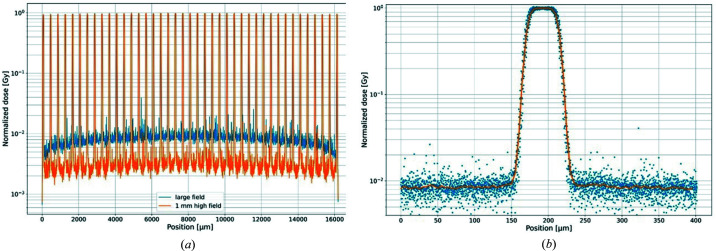
Results from the Monte Carlo simulation. Horizontal dose profiles of (*a*) the microbeam array and (*b*) a single microbeam.

Fig. 3 ▸(*a*) shows the horizontal dose profile of the entire microbeam field. The orange line represents the horizontal profile of a vertically 1 mm (FWHM) high synchrotron beam. When the sample is scanned through this beam the blue dose distribution is obtained (scanned field). As also observed in the measured beam intensity depicted in Fig. 2 ▸(*c*), due to scattered photons in the scanned field, the valley dose in the centre of the field is higher than at the edges. The right panel [Fig. 3(*b*)] shows an overlaid horizontal dose profile of a single microbeam in the scanned field. The orange line represents the averaged profile. The simulation yielded a PVDR of 123 ± 6.

**Figure 4:**
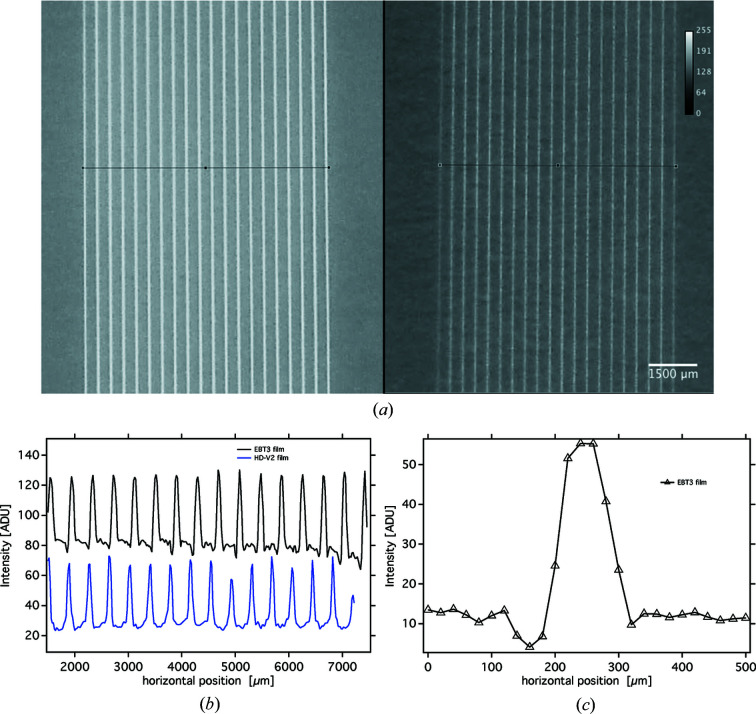
(*a*) Microphotographs of Gafchromic films EBT3 (left) and HD-V2 (right); (*b*) and (*c*) associated horizontal intensity profiles of the microbeam array.

Photographs of the exposed EBT3 and HD-V2 Gafchromic films were loaded into *ImageJ* (Schindelin *et al.*, 2012 ▸) and are presented in Fig. 4 ▸(*a*).

Following the description by Ocadiz *et al.* (2019 ▸), the peak dose was defined as the region 20 µm on both sides of the centre of the microbeams. To derive the peak dose, microphotographs of three FoVs were obtained and at least two 40 µm × 100 µm regions in the centre of the microbeams were used for analysis. The images were analyzed using the Histogram function of *ImageJ*. Mean intensity values were obtained and correlated to two types of calibration curve. One calibration curve was obtained at the synchrotron source and one calibration curve was obtained from films exposed at a conventional orthovoltage source at 100 kV (X-beam, Varian). The latter was done for the purpose of fine tuning, while the calibration curve obtained at the synchrotron contained only five dose values and therefore allowed only a coarse assessment of the dose obtained within a single microbeam

Horizontal plot profiles were created along the horizontal lines orthogonal to the direction of the microbeams. The resulting profiles, which had been averaged vertically over a total length of 2 mm, are shown in Fig. 4 ▸(*b*). Fig. 4 ▸(*c*) shows the horizontal intensity profile of one of the microbeams in the centre of the microbeam array recorded on the EBT3 film, again vertically averaged over a length of 2 mm. Conversely to Figs. 2 ▸(*c*) and 2(*d*) and probably due to their limited spatial resolution and dynamic range, neither of the Gafchromic films were able to resolve the increased intensity feature at the right-hand side (falling edge) of the microbeam.

Based on the EBT3 Gafchromic film measurements, the peak dose was approximately 65 Gy and the valley dose between 0.5 Gy and 0.75 Gy. Considering known material-dependent uncertainties with Gafchromic film dosimetry, which could be >10% with regards to energy-dependence and dose rate response, the results of the film dosimetry are in the same range as those obtained by Monte Carlo simulation and as measured by the sCMOS imager.

### 3.2. Irradiation of cell cultures   

**Figure 5:**
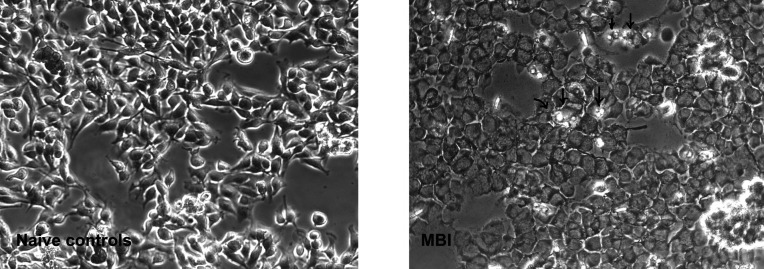
Naïve cell cultures before and after MBI (unstained cells, optical phase contrast, one microscopy FoV each). Blebbing of cells dying after irradiation can be seen (arrows).

**Figure 6:**
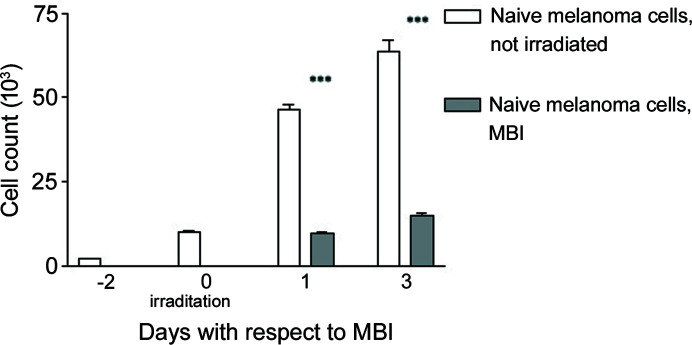
Melanoma cell count at 24 h and 72 h after MBI.

Using optical microscopy in phase contrast mode, the cells appeared flattened after MBI, possibly a sign of exsiccation after the disturbance of the membrane function. There was visible blebbing as a sign of impending disintegration of cells (Fig. 5 ▸). At 24 h after MBI, the melanoma cell numbers were reduced significantly (*p* = 0.0001) to approximately 20.5%, compared with not-irradiated naïve cells (Fig. 6 ▸). At 72 h after MBI, cell numbers were reduced to approximately 23.5% with MBI, compared with not-irradiated naïve cells. Although this was still a highly significant reduction of melanoma cell numbers (*p* = 0.0007), the surviving cell fraction increased again after irradiation.

One single fraction of MBI in the monoplanar technique from one single port has significantly reduced the number of melanoma cells compared with untreated cells. The *p* values are 0.0001 for 24 h and 0.0007 for 72 h after MBI (error bars for SEM, with *** marking high significance).

## 4. Discussion   

As part of the worldwide efforts in microbeam research, experiments in Europe were previously conducted exclusively at the biomedical beamline ID17 of the ESRF. This is the first time that an array of microbeams with the aim to irradiate a biological sample was produced at the biomedical beamline SYRMEP of the Elettra-Sincrotrone Trieste. Differences in technical parameters influencing the quality of the X-ray beam include the ring energy and the X-ray energy range. At the ESRF, but also at SPring-8 and the Australian Synchrotron, both of these parameters are significantly higher than at the Elettra-Sincrotrone Trieste.

Resulting from the lower beam energy at SYRMEP, we found a stronger horizontal angular beam divergence at the collimator position than at ID17. The maximal horizontal beam divergence at SYRMEP is 7 mrad. Lateral of the 20 most central collimator slits, the microbeams generated by the MSC became increasingly narrow due to the incoming beam angle. Therefore, not all available collimator slits could be used for our experiment, but only those in the centre of the SYRMEP beam. As a consequence, we recommend a different collimator design for future microbeam work at SYRMEP. This design should take into account the specific beam geometry and the lower energy of the incoming beam. The collimator design could consist of two sets of lamellae of which the more proximal one would follow the beam divergence. The resulting inhomogeneous dose distribution across the array could be compensated by inserting a lens or filter distal into the second set of microslits. The drawback of such a solution would be a slightly lower dose rate.

Irradiation in microbeam technique generates a non-homogeneous dose distribution in the target tissue and in the normal tissue in the path of the microbeam array. The dose distribution pattern is characterized by a PDVR. Depending on the energy spectrum and the density of the tissue, the PVDR decreases with increasing tissue depth.

If we assume that the peak dose, valley dose and resulting ratio of both determine the unique therapeutic effect of microbeam irradiation, both the achievable dose rate and the target depth related to the surface will determine the suitability of an X-ray source for MRT.

In our first pilot experiment for a microbeam array of 50 µm beam width and a centre-to-centre-distance of 400 µm, we obtained only a dose rate of approximately 70 Gy s^−1^. Compared with dose rates of several hundred Grays per second obtainable at the biomedical beamline of the Australian Synchrotron and several thousand Grays at ID17 of the ESRF, this appears to be very little. Given the technical prerequisites, the application of the same high peak dose will require a longer time at SYRMEP compared with both the Australian biomedical beamline and the ID17 at ESRF.

In tissues with characteristic physiologic movement, caused by heart beat-synchronous pulsation of tissue and breathing, a high dose rate is essential in order to apply high peak doses in a very short time. This will limit smearing of the beam edges caused by the physiologic movement. A sharp dose decrease at the microbeam edge is a prerequisite of a high PVDR, which in turn correlates to a high normal tissue tolerance. However, apart from the practical aspect that the risk of decreasing the PVDR subsequent to physiologic movement increases with the duration of the dose delivery time, there is very little knowledge about how fast dose deposition should be in order to be biologically efficient. Thus, especially in superficial tumours of the extremities, where tissue movement would be negligible, MBI might succeed also at lower dose rates than those available at the ESRF. It has been shown in pre-clinical research and veterinary studies that the so-called FLASH effect, following the delivery of a target dose at dose rates significantly higher than in conventional radiotherapy, results in significantly lower normal tissue damage and, at the same time, equal or even better tumour control (Favaudon *et al.*, 2014 ▸; Vozenin *et al.*, 2019 ▸; Chabi *et al.*, 2020 ▸). A dose rate of 40 Gy s^−1^ was sufficiently high to produce this effect (Favaudon *et al.*, 2015 ▸). Therefore, although the dose rate for MRT at SYRMEP was significantly lower than that achievable at the ESRF or the Australian Synchrotron, with the dose rate of 70 Gy s^−1^, obtained in our feasibility study, we would still be taking advantage of this FLASH effect.

Where superficial tumours like melanoma or osteosarcoma are targeted, as opposed to tumours of the central nervous system, working at lower energies might even be advantageous for the dose build-up. A slightly longer duration of the dose deposition in a superficial tumour of a limb, which would still be achieved within seconds, might be of no negative consequence, as far as the tumour regression is concerned. It is true that overall survival for patients with such malignant diseases is primarily determined by the extent of metastatic disease. However, there are at least two good reasons why it is nevertheless recommended to aim for the best possible control of the primary tumour: first of all, the recommended therapeutic approach for therapy-resistant sarcoma, for instance, is amputation of the affected extremity. Thus, if the need for amputation of the affected limb can be obviated by MRT, the result would clearly be an increase in the quality of life for these patients. Secondly, it can be expected that a better control of the primary tumour will reduce metastatic spread, in which case we even might see an increase in overall survival times.

We have shown that already with one single fraction of monoplanar unidirectional MBI where the microbeam array originated from a single port and with peak doses of approximately 65 Gy, a reduction of viable melanoma cell mass by almost 80% can be achieved.

A slight increase in cell numbers is seen between the 24 h and 72 h cell counts, suggesting that clonogenic tumour cells have survived the one single fraction of MBI with a peak dose of 65 Gy. This would be equivalent to a clinical situation where sufficient viable tumour cells are left to cause a regrowth/recurrence of the tumour. Therefore, from a clinical perspective, it is important to develop a strategy to achieve 100% melanoma cell destruction in order to prevent tumour recurrence.

Possible approaches for future studies would include the addition of a second coplanar MBI fraction, with a second microbeam array set orthogonal to the first MBI fraction, either immediately after or 24 h after the first irradiation. Equally interesting would be a combination of MBI and conventional radiotherapy, where MBI would be used as an integrated boost, to simulate what might be done in a human patient with a malignant tumour. Based on the highly significant reduction of tumour cells seen in our study, one would expect to see a much better response to broad beam irradiation if the MBI boost precedes conventional radiotherapy, because the number of viable tumour cells (tumour load) is already significantly reduced by MBI.

## 5. Summary   

In summary, we have shown that it is technically possible to generate arrays of quasi-parallel microbeams suitable for biomedical research at the biomedical beamline SYRMEP of the ELETTRA Sincrotrone Trieste. Taking into account the specific parameters of the SYRMEP beamline at the Sincrotrone Trieste, some of the technical solutions to conduct MBI research will differ from those at higher-energy beamlines. MRT studies *in vitro* and in suitable animal models can be conducted at dose rates of 100 Gy s^−1^ or less, supporting the international development efforts for cutting-edge MBI research.
